# Polygenic risk score phenome-wide association study reveals an association between endometriosis and testosterone

**DOI:** 10.1186/s12916-023-03184-z

**Published:** 2023-12-05

**Authors:** Isabelle M. McGrath, International Endometriosis Genetics Consortium, Grant W. Montgomery, Sally Mortlock

**Affiliations:** The Institute for Molecular Bioscience, The University of Queensland, Brisbane, QLD 4072 Australia

**Keywords:** Endometriosis, Testosterone, PheWAS, Mendelian randomisation, Genetic, Ovarian cancer

## Abstract

**Background:**

Endometriosis affects 1 in 9 women, yet it is poorly understood with long diagnostic delays, invasive diagnoses, and poor treatment outcomes. Characterised by the presence of endometrial-like tissue outside of the uterus, its main symptoms are pain and infertility. Endometriosis often co-occurs with other conditions, which may provide insights into the origins of endometriosis.

**Methods:**

Here a polygenic risk score phenome-wide association study of endometriosis was conducted in the UK Biobank to investigate the pleiotropic effects of a genetic liability to endometriosis. The relationship between the polygenic risk score for endometriosis and health conditions, blood and urine biomarkers and reproductive factors were investigated separately in females, males and females without an endometriosis diagnosis. The relationship between endometriosis and the blood and urine biomarkers was further investigated using genetic correlation and Mendelian randomisation approaches to identify causal relationships.

**Results:**

Multiple health conditions, blood and urine biomarkers and reproductive factors were associated with genetic liability to endometriosis in each group, indicating many endometriosis comorbidities are not dependent on the physical manifestation of endometriosis. Differences in the associated traits between males and females highlighted the importance of sex-specific pathways in the overlap of endometriosis with many other traits. Notably, an association of genetic liability to endometriosis with lower testosterone levels was identified. Follow-up analysis utilising Mendelian randomisation approaches suggested lower testosterone may be causal for both endometriosis and clear cell ovarian cancer.

**Conclusions:**

This study highlights the diversity of the pleiotropic effects of genetic risk to endometriosis irrespective of a diagnosis of endometriosis. A key finding was the identification of a causal effect of the genetic liability to lower testosterone on endometriosis using Mendelian randomisation.

**Supplementary Information:**

The online version contains supplementary material available at 10.1186/s12916-023-03184-z.

## Background

Endometriosis is a poorly understood common disease characterised by the growth of endometrial-like tissue outside of the uterus. The diagnostic delay for endometriosis is 7–11 years, which can be attributed to lack of disease awareness, variability in disease presentation, symptoms that overlap other conditions and the invasive nature of the diagnostic technique: laparoscopic surgery. There is a genetic component to endometriosis, with heritability estimates of 47–51% [1, 2], and the most recent genome-wide association study revealed 42 loci associated with the disease, which explain up to 5.01% of disease variance [3].

Whilst the flagship symptoms of endometriosis are pelvic pain and infertility, it is now appreciated endometriosis patients will often exhibit disturbances to multiple bodily systems beyond the reproductive system. Recently, a wealth of epidemiological data has identified many conditions that are comorbid with endometriosis, multiple of which have evidence of a shared genetic architecture [3–6]. An understanding of the overlapping traits with endometriosis is critical for comprehensive health management of the patient, for developing predictive tools, and for elucidating the underlying biology of endometriosis.

This study expands on previous approaches to characterising the overlap of endometriosis with other traits. A phenome-wide association study (PheWAS) is a technique whereby the association of an array of traits with a particular genetic variant is determined. This approach can be extended to study the association of multiple traits with the genetic liability to a trait of interest through use of the polygenic risk score (PRS) for the trait of interest, a PRS-PheWAS. Compared to analysis with disease status, use of the PRS for cross-trait analysis does not require a cohort with the presence of a trait of interest accurately ascertained. This is advantageous as many endometriosis cases may be undiagnosed and thus be present in control cohorts given the heterogeneity in symptom severity and the invasive and lengthy diagnosis process for endometriosis. Further, as a PRS-PheWAS does not use disease status, it specifically looks for pleiotropic effects of the disease-associated genetic variants, meaning the effects of a genetic liability to endometriosis can be studied in individuals without the disease.

## Methods

**Fig. 1:**
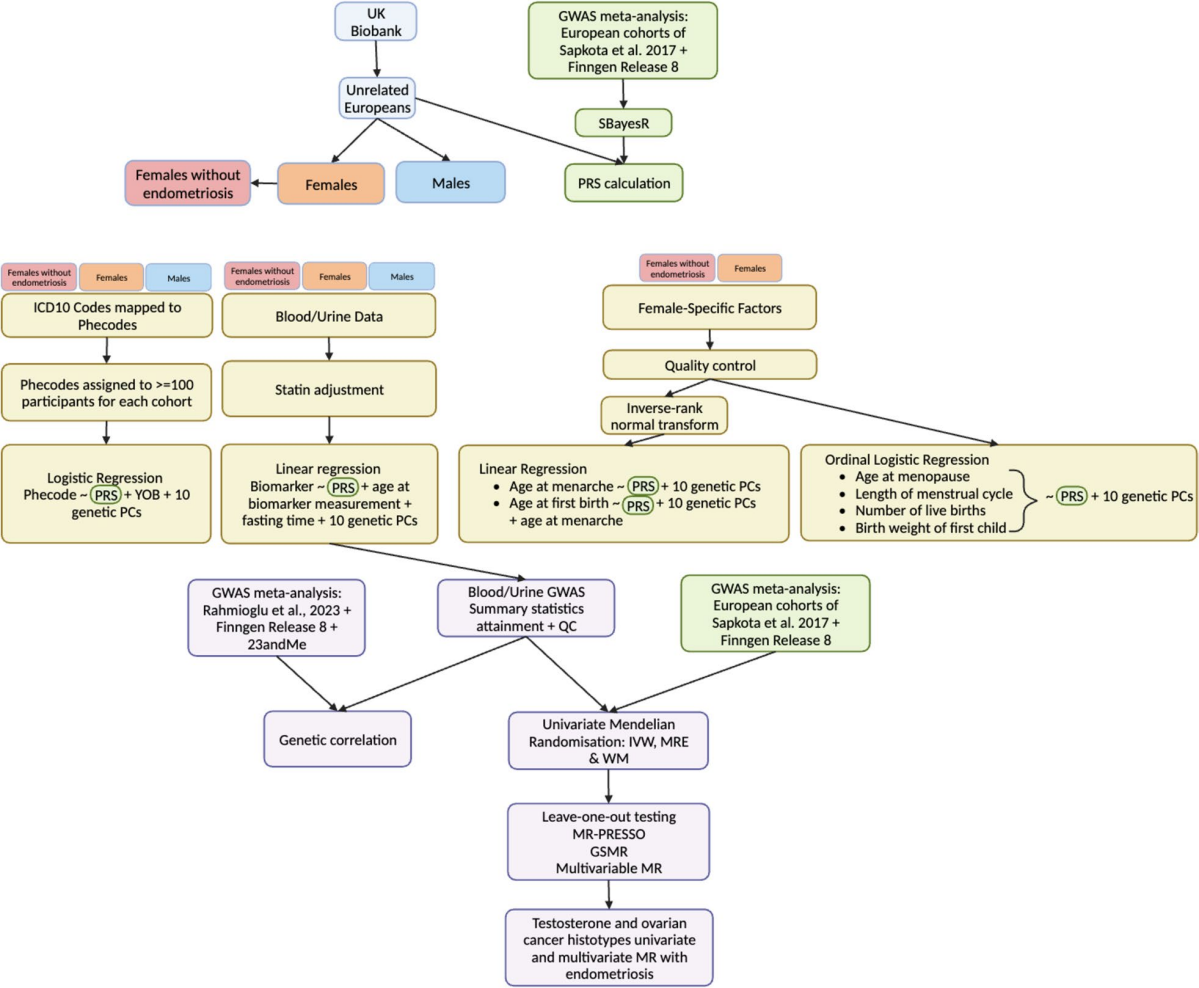
Flow diagram of methodology. PRS: polygenic risk score; PCs: principal components; GWAS: genome-wide association study; QC: quality control; MR: Mendelian randomisation. Created with Biorender.com

A flow diagram summarising the workflow and methods is employed in Fig. 1.

### Phenotype data from UK Biobank

The UK Biobank (UKB) is a large population database containing comprehensive health records and genetic data of approximately 500,000 individuals. The matched phenotype and genotype information allows use of the UKB for a PheWAS. Three groups of phenotype data were utilised: ICD10 diagnostic data, blood and urine biomarker data and female-specific factors. ICD10 diagnostic codes were mapped to phecodes and grouped into categories using the map from https://phewascatalog.org/files/Phecode_map_v1_2_icd10_beta.csv.zip and https://phewascatalog.org/files/phecode_definitions1.2.csv.zip. Where an ICD10 code mapped to multiple phecodes, one combination was selected. Phecode categories included infectious disease, neoplasms, endocrine/metabolic, haematopoietic, mental disorders, neurological, sense organs, circulatory system, respiratory, digestive, genitourinary, dermatologic, musculoskeletal, congenital abnormalities, symptoms, injuries and poisonings and other. Numerical data from the blood biochemistry and urine assays were collated for each participant. In total, there were 34 blood/urine variables available. Blood/urine biomarker data were log transformed prior to PheWAS analysis. The female-specific factors age at menarche, age at menopause, length of menstrual cycle, number of live births, birth weight of first child and age at first birth were analysed in the PheWAS. For some UKB participants, there were multiple datapoints for these female-specific factors due to follow-up visits. For number of live births, the last recorded value was considered to account for any live births proceeding the enrolment visit. For all other female-specific factors, the first recorded value was considered to minimise the recall period. Quality control was conducted to remove extreme values that may reflect pathologies or values with less than 10 participants: age at menarche between 8 and 20 and age at first live birth between 14 and 43 years were considered, menopause before 40 or after 63 years was excluded, menstrual cycle length was restricted to 22–36 days and birth weight of first child between 3 and 12 pounds was considered. Four or more live births were collapsed into a single category.

### Development of endometriosis PRS weightings

Summary statistics from seven European cohorts included in the Sapkota et al. 2017 meta-analysis (14,926 cases; 189,715 controls) [7] of endometriosis were meta-analysed alongside endometriosis GWAS summary statistics obtained from FinnGen Release 8 (13,456 cases, 100,663 controls). The meta-analysis was conducted in METAL using the classical approach with genomic control for each cohort. Although a more recent, better powered GWAS has been published [3], the current dataset was selected to avoid sample overlap between the data used to generate the SNP weightings for the PRS and the testing cohorts for the PRS. A Bayesian method, SBayesR [8], as implemented in GCTB 2.02, was used for adjusting the GWAS summary statistics effect sizes. SBayesR was performed with default settings, in addition to the exclusion of the MHC region, and imputation of the sample size.

### Calculation of PRS

Firstly, two subgroups of the UKB genotype data were curated: unrelated European males (*n* = 159,855) and unrelated European females (*n* = 188,221). Specifically, female sex was initially determined using the genotype data provided by UKB and confirmed by only including participants with Sex = Female in the phenotype data. Males had Sex = Male and Sex Genetic = Male in the phenotype data. Ancestry was determined using genetic information [9].

PRS for three cohorts: females, males and a sensitivity cohort comprising of females without an endometriosis diagnosis (*n* = 182,789) (henceforth referred to as the sensitivity cohort) were calculated using plink1.9’s score function [10] on the SBayesR weightings. The participants considered as endometriosis cases were determined by entries in 132,122–0.0 and 132,123–0.0 (Date and Source of N80 first report) and was further refined by excluding individuals with the ICD10 diagnostic code N80.0: endometriosis of the uterus, and no other endometriosis diagnosis (N80.1-N80.9). N80.0 refers to adenomyosis, which is currently recognised as a distinct disease to endometriosis.

### Running PheWAS

In a PheWAS, the association of a given genotype with multiple phenotypes is tested. This differs from a typical GWAS, where for a given phenotype, the association with multiple genotypes is determined. Here, the association of endometriosis PRS with multiple phenotypes was tested, i.e. a PRS-PheWAS, essentially testing whether genetic liability to endometriosis affects the probability of being diagnosed with other traits. PRS was converted to *z*-score for PRS-PheWAS.

The PRS-PheWAS was conducted using R’s glm function for logistic regression for the phecodes. The first 10 genetic principal components (PCs) and age (calculated using year of birth) were used as covariates. PCs were calculated on genotype data filtered to minor allele frequency > 5%, genotype missingness < 5%, SNPs passing Hardy–Weinberg exact test with a *p* value threshold of 1 × 10^−6^ and pruned for linkage disequilibrium (window size 50 kb, step size 5 variants, r^2^ threshold 0.2) for males and females, in addition to the female sensitivity cohort set. PCA was performed using plink2 PCA function with the approx flag [11]. Phecodes assigned to at least 100 participants were included in the PheWAS.

Linear regression using the lm function in R was used for the blood/urine data. Before running the PheWAS, certain biomarkers affected by statins were adjusted in individuals taking statins at enrolment. Adjustment for statin usage was possible for blood/urine biomarker data as statin usage at the time of biological sample collection is available in the UK Biobank. The codes for the medications recognised as statins were 1,141,146,234, 1,141,192,414, 1,140,910,632, 1,140,888,594, 1,140,864,592, 1,141,146,138, 1,140,861,970, 1,140,888,648, 1,141,192,410, 1,141,188,146, 1,140,861,958, 1,140,881,748 and 1,141,200,040. The adjustment method was based on a previously published GWAS of blood and urine biomarkers in the UKB [12]. Specifically, individuals who were taking statins at the first repeat visit, and not the enrolment visit, were identified. For each biomarker in these individuals, the ratio of on-statin biomarker value/pre-statin biomarker value was determined. The mean ratio across all individuals of each sex for each biomarker was determined. This was the sex-specific statin correction factor. Then to determine which biomarkers are affected by statin usage, participants taking statins at the first repeat visit only were utilised for a linear regression modelling the effect of each biomarker on the log ratio of pre-statin biomarker value to on-statin biomarker value. In this linear regression, covariates included Townsend Deprivation index, the first 10 PCs for all individuals of the corresponding cohort, age at enrolment and age difference between enrolment and first visit. For age, only the month and year were utilised. Traits that were significant (*P* < 0.05/34 as 34 blood/urine traits) in this linear regression analysis for males and females separately were flagged for adjustment in individuals taking statins at enrolment (Additional file 1: Table S1). Specifically, for individuals taking statins at enrolment, their value for biomarkers affected by statins was divided by the biomarker-specific statin correction factor. In females, apolipoprotein B, C-reactive protein, cholesterol, LDL direct and triglycerides were adjusted by the statin correction factor. In males, apolipoprotein B, C-reactive protein, cholesterol, direct bilirubin, LDL direct, microalbumin in urine, sodium in urine, testosterone and triglycerides were adjusted by the statin correction factor. In the PheWAS, fasting time, age at biomarker measurement (utilising month and year information) and the first 10 PCs were used as covariates.

For the female-specific factors, visual assessment for normality of a QQ plot on inverse-rank normal transformed data determined whether each variable should be treated as a continuous variable or an ordinal categorical variable. Age of menarche and age at first birth were treated as linear variables so tested with linear regression using R’s lm function, whilst age at menopause, length of menstrual cycle, number of live births and birth weight of first child were treated as ordinal categorical variables and tested with ordinal logistic regression using the polr function from the MASS package in R. Linear variables were inverse-rank normal transformed. As ordinal logistic regression does not output a *P* value, *P* values were calculated by comparing the *t*-values to a standard normal distribution. The first 10 genetic PCs were included as covariates for all female-specific factors. Age at menarche was also a covariate for age at first live birth.

Phenotypes were declared significant if they passed a stringent Bonferroni threshold (*P* < 0.05/n traits), whereby each set of traits (phecodes, blood/urine biomarker and female-specific factors) were considered separately. Analyses were conducted for the male cohort, female cohort and the sensitivity cohort.

### Genetic investigation of blood and urine biomarkers

The genetic relationship of biomarkers apolipoprotein A, apolipoprotein B, alanine aminotransferase, testosterone, bioavailable testosterone, triglycerides, HDL cholesterol, LDL cholesterol, albumin, calcium, sex hormone-binding globulin (SHBG) and urate with endometriosis was assessed. These traits were selected due to their significance or nominal significance in the PheWAS analysis and availability of appropriate GWAS summary statistics. SHBG was also included due to its previously reported strong correlation with testosterone. Bioavailable testosterone was also included, as most testosterone is bound to SHBG, and thus inactive. Summary statistics were downloaded from the GWAS Catalog (Table 1) [13]. GWAS summary statistics underwent quality control: missing SNP sample size was replaced with the published total sample size, rsIDs were updated to the rsID of the endometriosis summary statistics to ensure maximum SNP overlap, in the case of duplicated rsIDs the variant with the lowest *P* value was retained, and traits without SNP allele frequency had the allele frequencies of unrelated European females in the UKB appended. Two endometriosis datasets were utilised. In the first, utilised for genetic correlation, three endometriosis GWAS datasets were meta-analysed to maximise power: Rahmioglu 2023 (21,779 European ancestry cases, 449,087 European ancestry controls, 1713 Japanese ancestry cases, 1581 Japanese ancestry controls) [3], FinnGen release 8 (13,456 cases, 100,663 controls), and 23andMe, Inc. (4970 cases, 34,561 controls). SNP rsIDs were harmonised, SNPs with missing *P* or beta values were removed, SNPs with *P* < 0 or *P* > 1 were removed and in the case of duplicate SNPs, the SNP with the lowest *p* value was retained. The meta-analysis was conducted in METAL using the classical approach with genomic control for each cohort. The meta-analysis result underwent a secondary round of genomic control. As the allele frequency was not present in all three cohorts, the allele frequency from the largest cohort [3] was subbed in, restricting to SNPs present in this GWAS. The sample size was approximated by the sum of the cohorts the SNP was present in. The second endometriosis dataset, detailed earlier in the methods for the PheWAS, was the meta-analysis of the European component of the 2017 endometriosis GWAS [7] with FinnGen release 8 (13,456 cases, 100,663 controls). Although less powered, this second dataset was necessary to avoid sample overlap with the biomarker GWAS, as this is known to bias estimates for Mendelian randomisation. These GWAS summary statistics are referred to as dataset 2. Follow-up analysis of testosterone [14] and ovarian cancer utilised ovarian cancer summary statistics published in Phelan, Kuchenbaecker [15].

**Table 1:** GWAS Summary Statistics of Blood/Urine Biomarkers utilised for cross-trait analysis with endometriosis

Trait	Female-specific	Ancestry and sample size	Publication (GWAS Catalog Accession Number)
Apolipoprotein A	No	311,601 European ancestry individuals, 5550 African ancestry individuals, 6682 South Asian ancestry individuals	Sinnott-Armstrong, Tanigawa [12] GCST90019495
Apolipoprotein B	No	340,860 European ancestry individuals, 5962 African ancestry individuals, 7275 South Asian ancestry individuals	Sinnott-Armstrong, Tanigawa [12] GCST90019496
Alanine aminotransferase	No	342,387 European ancestry individuals, 6017 African ancestry individuals, 7325 South Asian ancestry individuals	Sinnott-Armstrong, Tanigawa [12] GCST90019492
Testosterone	Yes	230,454 European ancestry women	Ruth, Day [14] GCST90012112
Bioavailable Testosterone	Yes	188,507 European ancestry women	Ruth, Day [14] GCST90012102
Triglycerides (for Genetic Correlation)	No	1,320,016 European ancestry individuals	Graham, Clarke [16] GCST90239664
Triglycerides (for MR)	No	115,082 European ancestry individuals	Richardson, Sanderson [17] GCST90092992
Urate	No	342,087 European ancestry individuals, 6011 African ancestry individuals, 7328 South Asian ancestry individuals	Sinnott-Armstrong, Tanigawa [12] GCST90019524
HDL Cholesterol	No	313,372 European ancestry individuals, 5573 African ancestry individuals, 6689 South Asian ancestry individuals	Sinnott-Armstrong, Tanigawa [12] GCST90019510
LDL Cholesterol	No	341,875 European ancestry individuals, 6003 African ancestry individuals, 7319 South Asian ancestry individuals	Sinnott-Armstrong, Tanigawa [12] GCST90019512
Sex hormone-binding globulin	Yes	189,473 European ancestry women	Ruth, Day [14] GCST90012107
Albumin	No	313,032 European ancestry individuals, 5573 African ancestry individuals, 6687 South Asian ancestry individuals	Sinnott-Armstrong, Tanigawa [12] GCST90019493
Calcium	No	313,387 European ancestry individuals, 5576 African ancestry individuals, 6696 South Asian ancestry individuals	Sinnott-Armstrong, Tanigawa [12] GCST90019500

*MR* Mendelian randomisation

Genetic correlation assesses the average genome-wide correlation in variant effects between traits. The genetic correlation between endometriosis and the blood/urine traits was estimated using LDSC (v1.0.1) and precomputed LD scores from the 1000 Genomes European reference set. Using the munge_sumstats.py script, the alleles in the GWA summary statistics for each trait were crosschecked against HapMap3 SNPs used to estimate the LD scores using the merge-alleles function and chunk size 1,000,000. Genetic correlation was performed using the endometriosis dataset 1 GWAS summary statistics.

Mendelian randomisation analysis was performed to assess whether any of these traits could be linked to endometriosis via causality. Mendelian randomisation is a statistical technique that assesses causality through the use of genetic variants as instrumental variables (IVs) [18]. Initially, three models were applied: inverse-variance weighted (IVW), MR-Egger (MRE) and weighted median (WM). The IVW method assumes all variants satisfy the assumptions of MR. The MRE and WM methods are important sensitivity tests. MRE allows for an overall directional pleiotropic effect, which provides valid estimates if the pleiotropic effect on the outcome is independent of their effects on the exposure [19]. The WM method provides valid estimates if at least 50% of the variants are valid instrumental variables [20]. Heterogeneity and pleiotropy statistics were also calculated. Causality was assessed with endometriosis as both the exposure and outcome variable. SNPs were filtered using the MR Steiger directionality test to ensure they were more strongly associated with the exposure than the outcome. FDR-adjusted *P* values were calculated within each directional analysis (i.e. endometriosis as exposure, endometriosis as outcome), and within each MR method. Significant results (adjusted *P* < 0.05) were further investigated using additional MR methods GSMR [21] and MR-PRESSO. GSMR and MR-PRESSO detect and remove outlier SNPs, so the result is unlikely to be biased by pleiotropy. As many blood biomarkers show strong phenotypic correlation and have many shared genetic risk loci, multivariable MR was conducted for testosterone with SHBG, apolipoprotein A with HDL-C and triglycerides with LDL-C and apoliproprotein B. Multivariable MR determines the causal effect of an exposure on the outcome, conditional on the other exposures in the model. This is an appropriate sensitivity test to run when potential confounders are known. The TwoSampleMR package in R was utilised for both univariate and multivariable MR, whilst GSMR was performed as implemented in GCTA v1.94.1. The LD reference dataset for GSMR was the QIMRHCS cohort [7]. Independent SNPs with genome-wide significance (*P* < 5 × 10^−8^) were utilised as IVs. As many of the blood/urine biomarker summary statistics were derived from UK Biobank data, endometriosis dataset 2 summary statistics were utilised. All IVs were sufficiently powered: the F-statistics (beta^2^/se^2^) for each IV for every exposure were > 10.

## Results

### PRS-PheWAS results

**Fig. 2:**
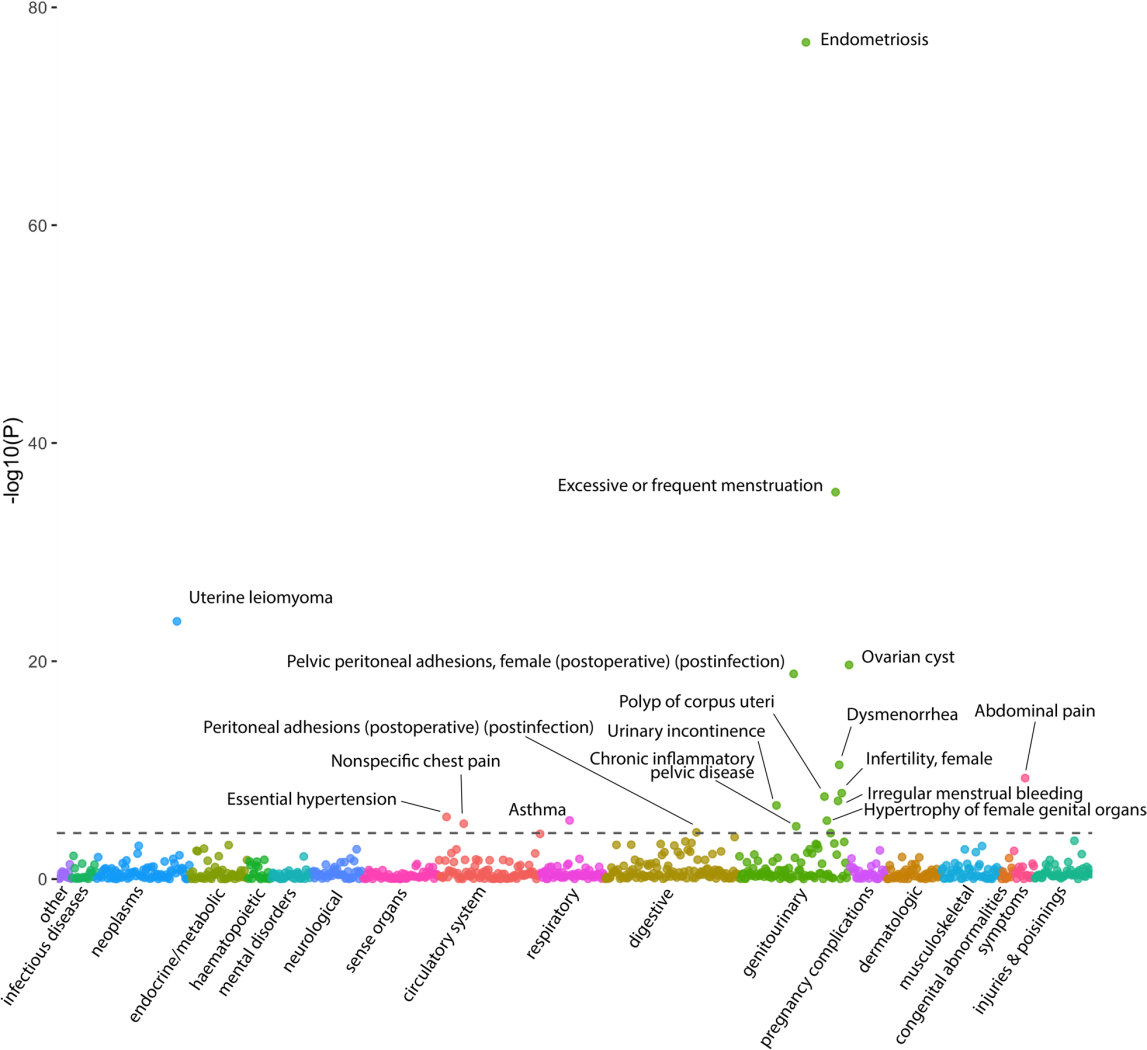
Female PRS-PheWAS for endometriosis in the UK Biobank. In total, 841 phecodes were tested for their association with endometriosis PRS. Traits are grouped and colour-coded into categories. The *P* value threshold for significance (dotted line) is 5.95 × 10^−5^ (Bonferroni-corrected threshold). The *P* values were generated from logistic regression, with the first ten genetic principal components and age as covariates. Traits with significant *P* values are annotated

The PheWAS of phecodes tested association of multiple phenotypes with endometriosis PRS. Phecodes aggregate multiple ICD10 codes corresponding to a similar phenotype. There were 17, 11 and 2 significant phecodes in the female, sensitivity and male cohort PheWASs, respectively (Additional file 1: Tables S2-S4). All significant phecodes were positively associated with endometriosis PRS. In the female phecode PheWAS, the top associated phecode was 615: Endometriosis (Fig. 2). Other highly associated traits included excessive/frequent menstruation, uterine leiomyoma, ovarian cyst, and pelvic peritoneal adhesions. All phecodes significant in the female analysis replicated at least nominal significance in the female sensitivity cohort, except for chronic inflammatory pelvic disease (*P* = 0.064). All 11 phecodes significant in the sensitivity cohort were also significant in the whole female cohort. The phecode for endometriosis was also significant in the sensitivity cohort. As the phecode definition of endometriosis includes the ICD10 code N80.0: endometriosis of the uterus, which here was excluded from the endometriosis definition, the endometriosis phecode in the female sensitivity cohort is representative of adenomyosis. In the male analysis, the two traits significantly associated with endometriosis PRS were abdominal pain and hyperplasia of prostate.

In the female-specific factor analysis, younger age at menopause, menarche and first live birth, and shorter length of menstrual cycle were significantly associated with endometriosis PRS (Table 2, Additional file 1: Table S5). This was replicated in the sensitivity cohort, although age at menopause was nominally significant (*P* = 0.047) (Additional file 1: Table S6).

**Table 2:** Female-specific factors associated with endometriosis PRS. Females in the UK Biobank were utilised. Estimate refers to the regression coefficient from linear regression (age at menarche and age at first live birth), or from ordinal logistic regression (age at menopause and length of menstrual cycle)

Biomarker	Estimate	SE	*P*
Age at menopause	− 0.016	0.005	2.64 × 10^−3^
Length of menstrual cycle	− 0.062	0.011	3.38 × 10^−8^
Age at menarche	− 0.017	0.002	4.72 × 10^−14^
Age at first live birth	− 0.021	0.002	1.08 × 10^−13^

SE Standard error, *P* *P* value

Multiple blood and urine biomarkers were associated with endometriosis PRS (Tables 3, Additional file 1: Tables S7-S9). All thirteen biomarkers significant in the female analysis replicated at the Bonferroni-corrected threshold or nominal significance in the female sensitivity cohort. In the male analysis five biomarkers were significant, four were also significant in the female analysis (triglycerides, HDL cholesterol, calcium, apolipoprotein A), whilst alkaline phosphatase had nominal significance in the female analysis.

**Table 3:** Significant blood/urine biomarkers associated with endometriosis PRS in females in the UK Biobank. Biomarkers were corrected for statin usage. Estimate refers to the regression coefficient from linear regression

Biomarker	Estimate	SE	*P*
Triglycerides	0.0140	0.0020	4.56E − 12
Calcium	0.0013	0.0002	2.56E − 08
Alanine aminotransferase	0.1396	0.0291	1.62E − 06
Urate	0.7117	0.1525	3.07E − 06
HDL cholesterol	− 0.0042	0.0009	6.37E − 06
Oestradiol	9.8021	2.3176	2.35E − 05
Gamma glutamyltransferase	0.3345	0.0794	2.53E − 05
Albumin	0.0265	0.0064	3.55E − 05
Apolipoprotein B	0.0022	0.0005	4.35E − 05
Testosterone	− 0.0060	0.0016	2.61E − 04
LDL direct	0.0065	0.0019	8.74E − 04
Apolipoprotein A	− 0.0022	0.0007	8.80E − 04
Total protein	0.0323	0.0100	1.23E − 03

*SE* Standard error, *P* *P* value

**Fig. 3:**
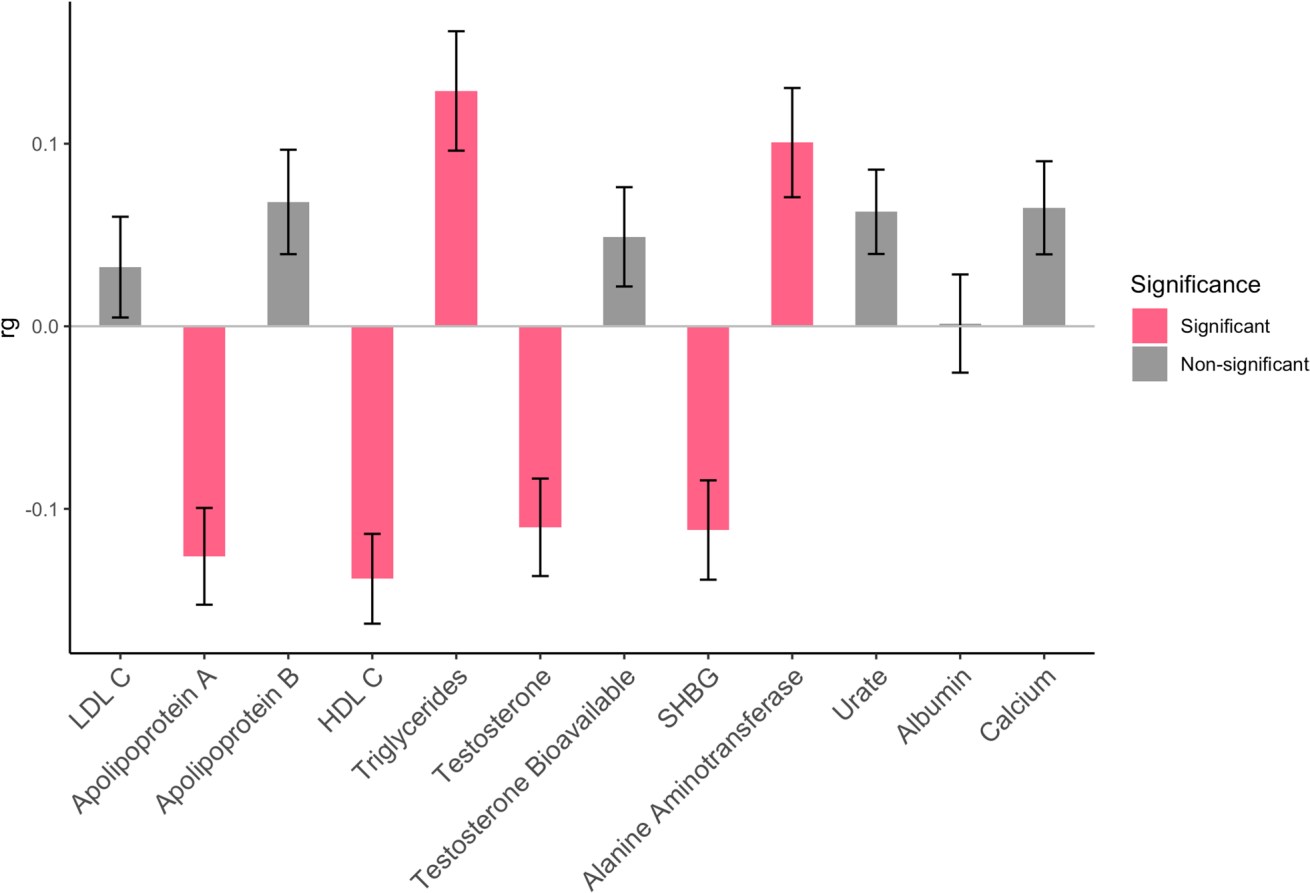
Genetic correlation of blood and urine traits with endometriosis. Genetic correlation (rg) was determined using LDSC. Traits were considered significantly genetically correlated with endometriosis if *P* < 4.17 × 10^−3^. Error bars represent standard errors

The relationship of endometriosis with a subset of the blood/urine biomarkers was further explored. Genetic correlation analysis revealed apolipoprotein A, HDL cholesterol, triglycerides, testosterone, SHBG and alanine aminotransferase were all significantly correlated with endometriosis and passed the stringent Bonferroni-corrected *P* value threshold (Fig. 3, Additional file 1: Table S10).

**Fig. 4:**
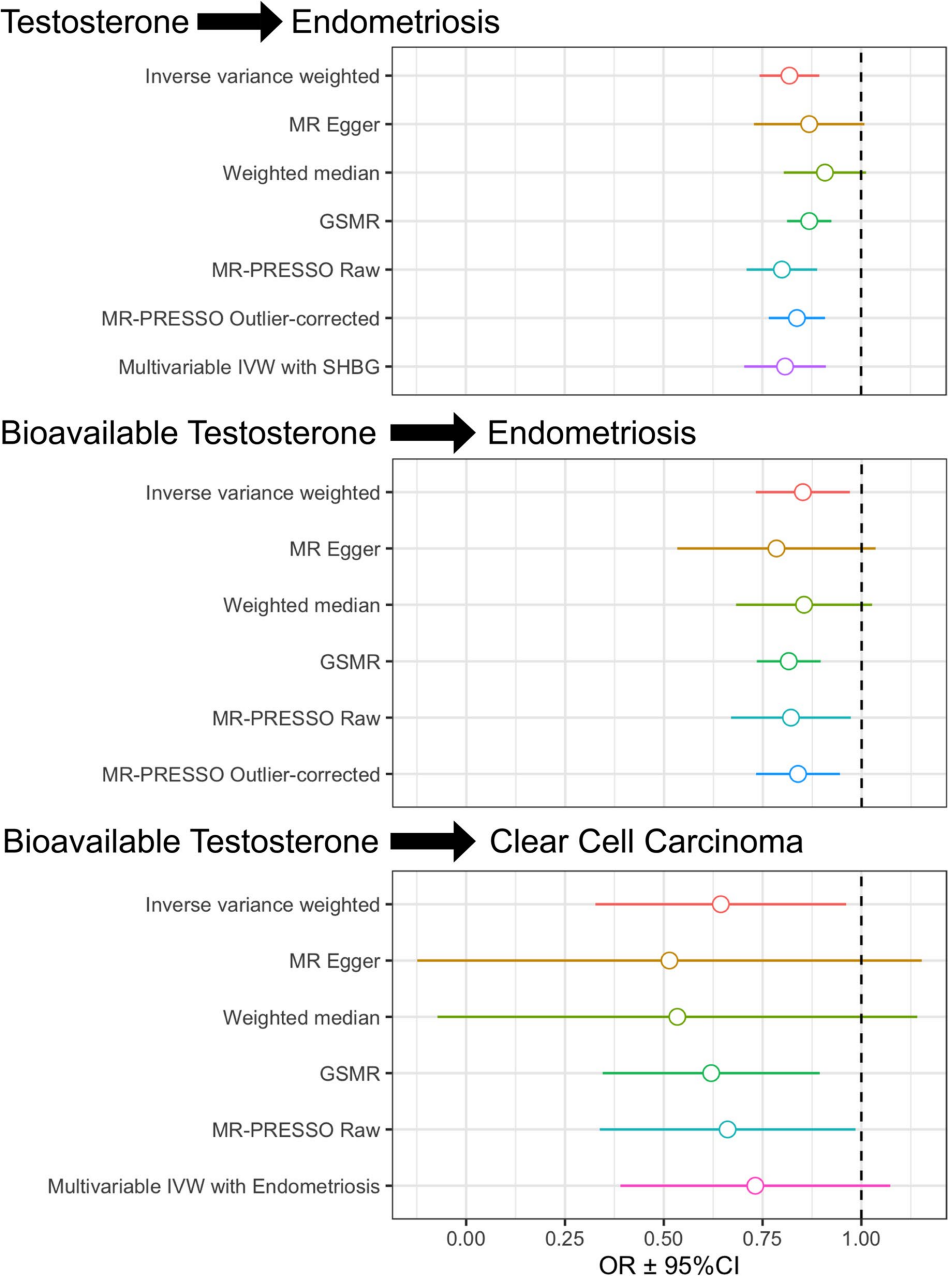
Causal relationships between testosterone, endometriosis and clear cell carcinoma ovarian cancer determined by Mendelian randomisation. A causal effect of testosterone and bioavailable testosterone on endometriosis is supported by multiple methods. A causal effect of bioavailable testosterone on clear cell carcinoma ovarian cancer was identified using univariate approaches, but adjustment for endometriosis using multivariable MR attenuated the effect and it was non-significant. MR: Mendelian randomisation, GSMR: generalised summary Mendelian randomisation, SHBG: sex hormone-binding globulin, IVW: inverse-variance weighted. MR-PRESSO: Mendelian Randomization Pleiotropy Residual Sum and Outlier

A negative causal effect of testosterone on endometriosis was supported by the IVW method (*b* =  − 0.20, *P* = 2.12 × 10^−7^, *P*_*adjusted*_ = 2.54 × 10^−6^) Fig. 4, Additional file 1: Table S11). Although heterogeneity was present, this relationship was not driven by any individual SNP, as indicated by leave-one-out analysis (Additional file 1: Table S12A). Whilst MRE and WM were not significant, the direction of effect was concordant with the IVW result, and the unadjusted MRE result was significant (*b* =  − 0.14, *P* = 0.049, *P*_*adjusted*_ = 0.26). Complementary tests MR-PRESSO and GSMR supported the result: the MR-PRESSO method was significant, even after adjustment for outliers (*b* =  − 0.18, *P* = 2.14 × 10^−6^), and GSMR was also significant (*b* =  − 0.14, *P* = 8.41 × 10^−7^). As the majority of testosterone is bound to SHBG and inactive, we also considered the effect of bioavailable testosterone on endometriosis. The IVW method for bioavailable testosterone was also significant with a negative causal effect (*b* =  − 0.16, *P* = 8.04 × 10^−3^) (Fig. 4). Although there was significant heterogeneity in the IVW model, the result was robust to leave-one-out analysis (Additional file 1: Table S12B). MR-PRESSO and GSMR supported a negative causal effect of bioavailable testosterone on endometriosis (MR-PRESSO outlier adjusted: *b* =  − 0.18, *P* = 1.53 × 10^−3^, GSMR: *b* =  − 0.20,* P* = 7.75 × 10^−7^). In a multivariable model including SHBG, the effect of overall testosterone on endometriosis was retained (*b* =  − 0.21, *P* = 5.03 × 10^−5^).

A causal effect of testosterone on ovarian cancer risk has previously been reported using MR approaches [14]. Likewise, a causal effect of endometriosis on ovarian cancer has also been reported [22]. Therefore, we investigated the causal pathways between these three traits. Firstly, we evaluated the causal effect of testosterone on ovarian cancer histotypes, as the previously reported analysis was limited to overall ovarian cancer. A negative causal effect of bioavailable testosterone was identified on one histotype: clear cell carcinoma ovarian cancer with the IVW method (*b* =  − 0.44, *P* = 6.61 × 10^−3^, *P*_*adjusted*_ = 0.046), in the absence of heterogeneity (Fig. 4, Additional file 1: Table S13). The unadjusted *P* values and direction of effect for the WM (*b* =  − 0.63, *P* = 0.0432) and MRE (*b* =  − 0.66, *P* = 0.0430) methods supported this result for bioavailable testosterone (Fig. 4, Additional file 1: Table S13). The IVW result was not driven by an individual SNP (Additional file 1: Table S12C). Both MR-PRESSO (*b* =  − 0.41, *P* = 0.014, no outliers detected) and GSMR (*b* =  − 0.48, *P* = 6.48 × 10^−4^) also supported the causal effect. Total testosterone also had concordant directions of effect on clear cell carcinoma (Additional file 1: Table S13). When considering the effect of bioavailable testosterone on clear cell ovarian cancer in a multivariable model with endometriosis, the causal effect of bioavailable testosterone was no longer significant (*P* = 0.073), whilst endometriosis retained its causative effect (*b* = 0.78, *P* = 1.80 × 10^−13^) (Fig. 4, Additional file 1: Table S14).

## Discussion

In this study, we use a PheWAS approach to identify conditions, female-specific traits, and blood/urine biomarkers associated with the genetic liability to endometriosis. A PheWAS approach differs from epidemiological and genomic studies through the integrated analysis of phenotype and genotype data. This approach enabled investigation of the effects of genetic liability to a disease in the absence of disease: utilisation of a female cohort without endometriosis diagnoses and a male cohort suggested that association of many traits with endometriosis cannot solely be attributed to the physical presence of endometriosis. Using Mendelian randomisation approaches, we also identify a possible causal effect of the genetic liability to lower testosterone on endometriosis and clear cell ovarian cancer, which, following further validation, may have important clinical implications for both endometriosis and ovarian cancer.

Given many endometriosis diagnoses are likely missed in the UK Biobank participants [23], the exclusion of individuals with an endometriosis diagnosis from the sensitivity cohort was likely imperfect. In addition to the endometriosis sensitivity cohort, males were used as a high-confidence endometriosis-free group to investigate the effects of genetic liability to endometriosis in the absence of the physical presence of endometriosis. There were two phecodes associated with the endometriosis PRS in males: abdominal pain and hyperplasia of prostate. The relationship with prostate hyperplasia is intriguing, as although the cause of benign prostate hyperplasia is unclear, inflammation and proliferation, also characteristic features of endometriosis, are key [24]. Given these individuals did not have a history of endometriosis, the association of abdominal pain with the endometriosis PRS must be explained by factors beyond presence of the lesion. A shared genetic background has previously been identified between endometriosis and multiple pain traits [3, 5]. Multiple other traits previously identified to have a shared genetic background with endometriosis were significant in the female analysis but not the male analysis. This may be explained through sex-specific hormonal-related pathways being involved in the overlap of endometriosis with these traits. Pain is an incentive to seeking an endometriosis diagnosis, so in addition to the endometriosis genetic risk signals capturing variants associated with lesion growth, gynaecological healthcare-seeking factors such as sensitivity to pain signals may also be captured. Therefore, the association of abdominal pain in males with the endometriosis PRS could be explained by the endometriosis risk variants being enriched for pain sensitivity signals. However, this explanation does not negate pleiotropic effects of the genetic variants on both endometriosis and pain sensitivity, or pain-causing effects of the endometriosis lesions, given most individuals report a reduction in pain in the short term following surgical removal of the lesions [25]. Disentangling the genetic effects of the risk signals for endometriosis on lesion characteristics and endometriosis symptoms will be of interest for future studies. Further, the association of traits with the endometriosis PRS in males and in the female sensitivity cohort suggests epidemiological studies should look for symptoms and traits in relatives of individuals with endometriosis, which could prove valuable disease-predictive factors.

The traits in the PRS-PheWAS analysis identified to be associated with endometriosis genetic risk are directly dependent on the characteristics of the cohorts used to generate the GWAS summary statistics used to calculate the PRSs. Endometriosis is a highly heterogenous condition, with a large spectrum in symptom intensity. Further, women face a multitude of barriers in accessing a diagnosis including the trivialisation of their symptoms, the non-specificity of their symptoms, cost of seeking healthcare and the invasiveness of the gold-standard diagnosis (laparoscopic surgery). Therefore, women with more severe symptoms and fewer barriers to seeking healthcare are more likely to be diagnosed, and thus present in the case sample of a GWAS study. Likewise, there are likely many undiagnosed cases in the control cohort, reducing the power of the GWAS. If there is variability in the genetic architecture of endometriosis that is correlated with healthcare-seeking factors, the results of the PRS-PheWAS analysis may be restricted to a subset of endometriosis cases.

A key finding of this study was the suggestion of a causal effect of genetically predicted lower testosterone on endometriosis using MR. This effect was consistent across multiple models, and when using multiple measures of testosterone: overall testosterone and bioavailable testosterone. Although not all MR models were significant, the direction of effect was consistent between models. A limitation of this approach to modelling the relationship with the MR techniques utilised is that a linear effect is assumed: meaning the model suggests lower testosterone is causative, and higher testosterone is preventative of endometriosis. However, this may be a simplification of the relationship if testosterone only exerts its causative/preventative effect in one of these directions. The testosterone SNPs used for MR were female-specific which is important given there is no genetic correlation between testosterone in males and females [14]; however, they were generated from a GWAS of mostly postmenopausal adults. Given endometriosis onsets in the early reproductive years, assessment of the causative effect using adolescent, childhood and/or prenatal testosterone-associated SNPs should be assessed if the genetic control of testosterone differs at these stages.

Nevertheless, a role for alterations to the hypothalamic-pituitary–gonadal (HPG) axis, and a role for testosterone, specifically lower prenatal testosterone in endometriosis, has been previously discussed [26–28]. Differences in anogenital distance, a proxy for prenatal testosterone, between endometriosis cases and controls implicates lower prenatal testosterone in endometriosis cases [29]. In females, testosterone is synthesised from cholesterol in the adrenal gland and ovaries, and the primary regulator of testosterone levels in females is this steroid biosynthesis pathway [30]. In the ovary, testosterone is converted to oestradiol by aromatase, which is under the control of follicle-stimulating hormone (FSH). Elevated FSH has been reported in endometriosis cases (although some studies find no difference), alongside increased oestradiol only in the menstrual fluid (not in circulation) and increased aromatase activity in the eutopic endometrium [27]. In the PheWAS, oestradiol levels were positively correlated with endometriosis PRS; however, this should be considered cautiously as most women in the UKB are postmenopausal. In contrast, a reduced ovarian oestrogen to testosterone ratio, reduced FSH and reduced ovarian aromatase activity are observed in polycystic ovarian syndrome (PCOS). Alternate alleles of SNPs in strong LD upstream of *FSHB* are alternatively associated with endometriosis and PCOS: i.e. one haplotype confers risk to endometriosis, the other to PCOS [31].

There are a few possible mechanisms of lower testosterone impacting endometriosis risk. In support of Sampson’s retrograde menstruation hypothesis for endometriosis, lower prenatal and postnatal testosterone cause earlier menarche, shorter menstrual cycles and thicker endometrial lining, increasing the exposure to menstruation [27]. Oestrogens have inflammatory effects, whilst androgens have anti-inflammatory effects, so a high oestrogen:testosterone ratio could promote inflammation in response to ectopic endometrial tissue [27]. The Müllerian remnants are another hypothesis for endometriosis, whereby misplaced stem cells are activated by a stimulus [32, 33]. Low testosterone could contribute to the activation of these stem cells by facilitating a high inflammatory environment, and/or through facilitating deposition of these stem cells [27]. Testosterone is a controller of HOXA10 expression [34], which is a key player in the development of the female reproductive tract, and has been implicated in endometriosis [35]. Low testosterone could also contribute to pain in endometriosis, through mechanisms such as the inverse association with inflammation, and/or through links with β-endorphin levels within the central nervous system [28]. Importantly, a role for prenatal testosterone and early disturbance of the HPG axis in endometriosis would imply a developmental origin for endometriosis [28].

Previously a causative effect of lower testosterone on ovarian cancer was reported using MR [14]. Analysis of the major ovarian cancer histotypes indicated this causative effect was restricted to clear cell carcinoma; however, this effect was attenuated and non-significant when considered in a multivariable MR model with endometriosis. This suggests the effect of lower testosterone on clear cell carcinoma may be partially mediated through endometriosis; however, confidence intervals were large, so validation in larger datasets is necessary. Endometriosis has been previously identified as a cause of multiple histotypes of ovarian cancer: most strongly clear cell carcinoma and endometrioid ovarian cancer [22]. As lower testosterone did not show a causative effect on endometrioid ovarian cancer, testosterone may play a role in determining the histotype of ovarian cancer resulting from the endometriosis. Interestingly, polycystic ovarian syndrome, for which high testosterone is a characteristic feature and identified as causative using MR [14], has been determined as preventative for endometrioid ovarian cancer using MR techniques [36].

Four female-specific factors were associated with the endometriosis PRS: earlier age at menopause, shorter length of menstrual cycle, earlier age at menarche and earlier age at first live birth. A significant genetic correlation of endometriosis with these traits has previously been reported [3]. Shorter length of menstrual cycle and earlier age at menarche in endometriosis, also observed in epidemiological data, mean a greater exposure to menstruation, in support of Sampson’s theory of retrograde menstruation for endometriosis [37, 38]. The association of earlier age at first live birth, a proxy for fertility, with endometriosis PRS likely owes to the progressive nature of fertility issues. Age at first birth remained significant in the female sensitivity cohort. This may be explained by the presence of undiagnosed endometriosis cases in this cohort, and/or the direct effects of some endometriosis risk loci on infertility. Well-designed epidemiological studies of the relationship between age of menopause and endometriosis are lacking [39]. One study reported an increased risk of early natural menopause (< 45 years) in endometriosis patients [40]. Potential mechanisms include effects of endometrioma (endometriosis on the ovary) on ovarian function, effects of surgical excision of endometrioma [41] and effects of endometriosis-related traits such as reduced body mass index [39]. The attenuation of the effect of endometriosis PRS on age at menopause when endometriosis cases were excluded may point to effects of lesion presence on age at menopause, but further validation is needed.

The association of blood biomarkers with the endometriosis PRS provides promising evidence these biomarkers could be useful in predicting endometriosis. However, as the biomarker measurements from the UKB utilised here were measured in women older than the typical age for seeking an endometriosis diagnosis, their prediction accuracy should be assessed in a younger cohort, prior to surgical excision of the endometriosis lesions. Given most women in the UKB are postmenopausal, this is particularly relevant for biomarkers that are strongly affected by menopause, such as oestrogen. The association of various lipid-related biomarkers aligns with the association of endometriosis with cardiovascular traits in the phecode PheWAS and has been previously reported in epidemiological data [42]. Triglycerides show significant differences between endometriotic and normal endometrium of endometriosis patients [43]. The absence of any causal relationships between endometriosis and most blood/urine biomarkers may suggest pleiotropic genetic effects and/or non-genetic factors may be responsible for the overlap. One limitation of the genetic analysis is that for most traits the GWAS summary statistics were not female-specific, nor specific to young reproductive aged women. Timely and female-specific genetic associations may be necessary to reveal causal relationships and the true magnitude of genetic overlap with endometriosis. Further, not all GWAS summary statistics were derived from a purely European ancestry sample; however, the non-European component is small, and thus any bias is expected to be small.

## Conclusions

We have performed a comprehensive PRS-PheWAS for endometriosis. Associations of traits with genetic liability to disease, rather than disease presence, has provided interesting insights into the comorbidity of these traits with endometriosis and has ramifications for co-treatment of these diseases. Validation of a causal effect of lower testosterone on endometriosis using MR, and the finding of an effect of lower testosterone on clear cell carcinoma, prompts further investigation into the developmental origins of endometriosis and the malignant transformation of endometriosis into ovarian cancer in relation to testosterone.

### Supplementary Information

**Additional file 1:** **Table S1.** Statin Adjustment Factors and Linear Regression Analysis of Effect of Statin Usage on Biomarker Levels. **Table S2.** PRS-PheWAS of phecodes in females utilising endometriosis PRS. **Table S3.** PheWAS of phecodes in females without endometriosis utilising endometriosis PRS. **Table S4.** PheWAS of phecodes in males utilising endometriosis PRS. **Table S5.** PheWAS of female-specific factors in females utilising endometriosis PRS. **Table S6.** PheWAS of female-specific factors in females without endometriosis utilising endometriosis PRS. **Table S7.** PRS-PheWAS of blood/urine biomarkers in females utilising endometriosis PRS. **Table S8.** PRS-PheWAS of blood/urine biomarkers in females without endometriosis utilising endometriosis PRS. **Table S9.** PRS-PheWAS of blood/urine biomarkers in males utilising endometriosis PRS. **Table S10.** Genetic Correlation (rg) between endometriosis and blood/urine biomarkers. **Table S11.** Mendelian randomisation of endometriosis and blood/urine biomarkers. **Tables S12.** A-C Leave one out analyses. **S13.** Mendelian randomisation ovarian cancer and testosterone. **S14.** Multivariable Mendelian randomisation.

## Data Availability

Data can be accessed from UK Biobank (https://www.ukbiobank.ac.uk/) as per their published data access procedures. Summary data for the FinnGen Endometriosis GWAS is available from their results portal (https://www.finngen.fi/en/access_results). Publicly available GWAS summary data is cited in Table 1 or detailed in text. Endometriosis GWAS summary statistics, where not publicly available, are available on request. Any additional data supporting the conclusions of this article are included within the article and its additional files.

## Abbreviations

FSH: Follicle-stimulating hormone
GSMR: Generalised summary Mendelian randomisation
HPG: Hypothalamic-pituitary–gonadal
IVs: Instrumental variables
IVW: Inverse-variance weighted
MRE: MR-Egger
MR-PRESSO: Mendelian Randomization Pleiotropy Residual Sum and Outlier
PCOS: Polycystic ovarian syndrome
PCs: Principal components
PheWAS: Phenome-wide association study
PRS: Polygenic risk score
SHBG: Sex hormone-binding globulin
UKB: UK Biobank
WM: Weighted median
